# Screening of polycyclic aromatic hydrocarbons in multiple fish species and common whelk in the Faroe Islands using a modified QuEChERS method

**DOI:** 10.1007/s11356-025-37058-z

**Published:** 2025-10-21

**Authors:** Ziff Maria Kristensen, Maria Eckardt Manniche, Matteo Ottaviani, Jan H. Christensen, Peter Christensen, Sigurd Christiansen, Nikoline Juul Nielsen

**Affiliations:** 1 Department of Plant and Environmental Sciences, University of Copenhagen, Copenhagen, Denmark; 2 Danish Offshore Technology Centre, Technical University of Denmark, Kongens Lyngby, Denmark; 3 Faculty of Science and Technology, University of the Faroe Islands, Faroe Islands, Tórshavn, Denmark

**Keywords:** Anthracene, Bioindicator, Fish liver, Marine environment, Arctic pollution, Cod, Saithe, Sculpin

## Abstract

This study screened multiple fish species and marine common whelk to evaluate their potential as bioindicators of local pollution by polycyclic aromatic hydrocarbons (PAHs) in the Faroe Islands. Shorthorn sculpins (*Myoxocephalus scorpius*), Atlantic cod (*Gadus morhua*), common dab (*Limanda limanda*), saithe (*Pollachius virens*), and common whelk (*Buccinum undatum*), which all populate different tropic levels in the ecosystem were caught around Tórshavn Harbor, Runavík Harbor, Sund, Gamlarætt, and Kirkjubøur. The fish livers and the visceral complex of the common whelk were extracted and analyzed for the 16 PAHs regulated by the U.S. EPA using a modified QuEChERS method and gas chromatography-mass spectrometry. We detected and quantified acenaphthylene, fluorene, anthracene, phenanthrene, fluoranthene, and pyrene in the samples. Of the benthic species, common dab had the highest average ∑PAHs concentration of 11 ± 2 ng/g (ww) tissue. Sculpins had an average ∑PAHs concentration of 5 ± 2 ng/g (ww) liver. The highest concentration of PAHs, regardless of species, was found in Tórshavn with an average ∑PAH concentration of 12 ± 2 ng/g (ww). A principal component analysis showed that benthic species had a higher correlation to high molecular weight PAHs, whereas specific PAH concentration patterns for cods and saithe were less prevalent. Based on these results and the commonly agreed upon bioindicator criteria, the benthic species, common whelks, and sculpins were concluded to be the most suitable indicators of PAH pollution. Proper source identification was not possible due to low sampling size; however, assessing the PAH distribution pattern in the fish species together with the nearby human activities in the area indicate both pyrogenic and petrogenic origin with petrogenic sources being the main contributor.

**Graphical Abstract:**

1) Passive and active sampling in the target areas. 2) Dissection of fish and liver collection. 3) Homogenization and extraction with QuEChERS procedure. 4) GC–MS analysis and data treatment.

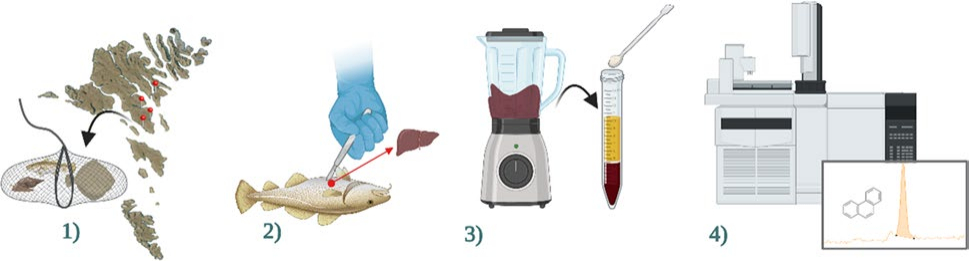

**Supplementary Information:**

The online version contains supplementary material available at 10.1007/s11356-025-37058-z.

## Introduction

Since the early 2000 s, Arctic and sub-Arctic areas such as the Faroe Islands have become easily accessible and appealing tourist destinations (Tourtellot 2007). The increase in human activities in the Faroe Islands has led to an increase in pollution of the marine environment by polycyclic aromatic hydrocarbons (PAHs) from local sources such as ship traffic, industry, and sewage effluents. Several PAHs are carcinogenic compounds, and toxicology studies have further identified signs of genotoxicity, immunotoxicity, endocrine disruption, and oxidative stress (Honda & Suzuki 2020). Due to their negative impact on health, the United States Environmental Protection Agency (U.S. EPA) has classified 16 PAHs as priority pollutants (U.S. EPA Appendix A part 423, December 2013), and the European Union has included them in their list of priority hazardous substances for surface waters in the Water Framework Directive (Directive 2000/60/EC). PAHs can either be of pyrolytic origin, produced by the incomplete combustion of organic materials, especially those that originate from fossils; or of petrogenic origin, spread in the environment from the discharge of petroleum components such as diesel, heavy fuel, lubricating oil, and coal products. PAHs consist of a variety of multiple conjugated rings and by examining the ring structure and their prevalence in the environment, a connection between the ring structure and their source of pollution can be established (Rocha & Palma 2019). The concentration ratios between low molecular weight PAHs (LMW PAHs) and high molecular weight PAHs (HMW PAHs) can be used to distinguish between petrogenic and pyrogenic sources (Olayinka et al. 2019; Tobiszewski & Namieśnik 2012).

Atmospheric deposition and petroleum spillage are the prominent sources of PAHs in the marine environment (Canedo-Lopez et al. 2020). PAHs that enter the aquatic ecosystem can adsorb fine-grained sediments and suspended particles, making marine sediments an effective sink and indicator for PAH pollution levels (Nemr et al. 2007). The monitoring of PAHs through marine organisms reflects the bioavailability and uptake mechanisms of PAHs by marine biota (Saunders et al. 2022). Fish and benthic organisms are exposed to PAHs via multiple routes including direct contact with polluted water through the gills, ingestion of prey or sediment, and through transdermal exposure to PAHs. The bio-accumulation pattern of PAHs varies in aquatic organisms depending on their trophic level, spatial distribution in the water column, habitat types, and feeding strategy (Baali et al. 2019).

A suitable bioindicator organism should accumulate pollutants to an extent that enables assessment of exposure, should be easy to sample, be stationary, and widely distributed (Conti & Cecchetti 2003). Benthic fish and invertebrates have therefore been used as bioindicators for environmental monitoring (Khan 2011; Snyder et al. 2015). However, detecting PAHs above the limit of detection in marine biota can be challenging, due to their binding rate on particulate matter but also due to different metabolization rates between marine vertebrates (Pampanin & Sydnes 2013). Species with high metabolism are therefore not reliable as bioindicators. Several studies have demonstrated that biliary PAH-metabolites are sensitive biomarkers for recent exposure (i.e., days or weeks) to PAHs (Beyer et al. 2010). In contrast, hydrophobic compounds, such as non-metabolized PAHs, found within animal tissues, such as liver and muscle, indicate longer exposure and accumulation period (e.g., weeks or months) (Rahmanpour et al. 2014). There are certain limitations related to the dynamic nature of bile (i.e., variable volume, composition, and concentration) together with technical challenges especially related to sampling, the metabolization rate, as well as the complex nature of PAHs metabolites. Less complex sampling and metabolization are associated with the use of marine vertebrate liver and tissue; the drawback of these matrices is however low analyte concentrations and high fat content (Struch et al. 2019).

A quick, easy, cheap, effective, rugged, and safe (QuEChERS) method can be used on wet matrices, requires just few grams of sample, and is therefore suitable for use in local laboratories. The aim of this study is to evaluate possible PAH pollution in marine biota in selected areas of the Faroe Islands and assess the best bioindicator from the available marine species. This will be done by implementation of the QuEChERS method followed by chemical analysis using GC–MS.

## Sampling

### Sampling area and sample handling

**Fig. 1:**
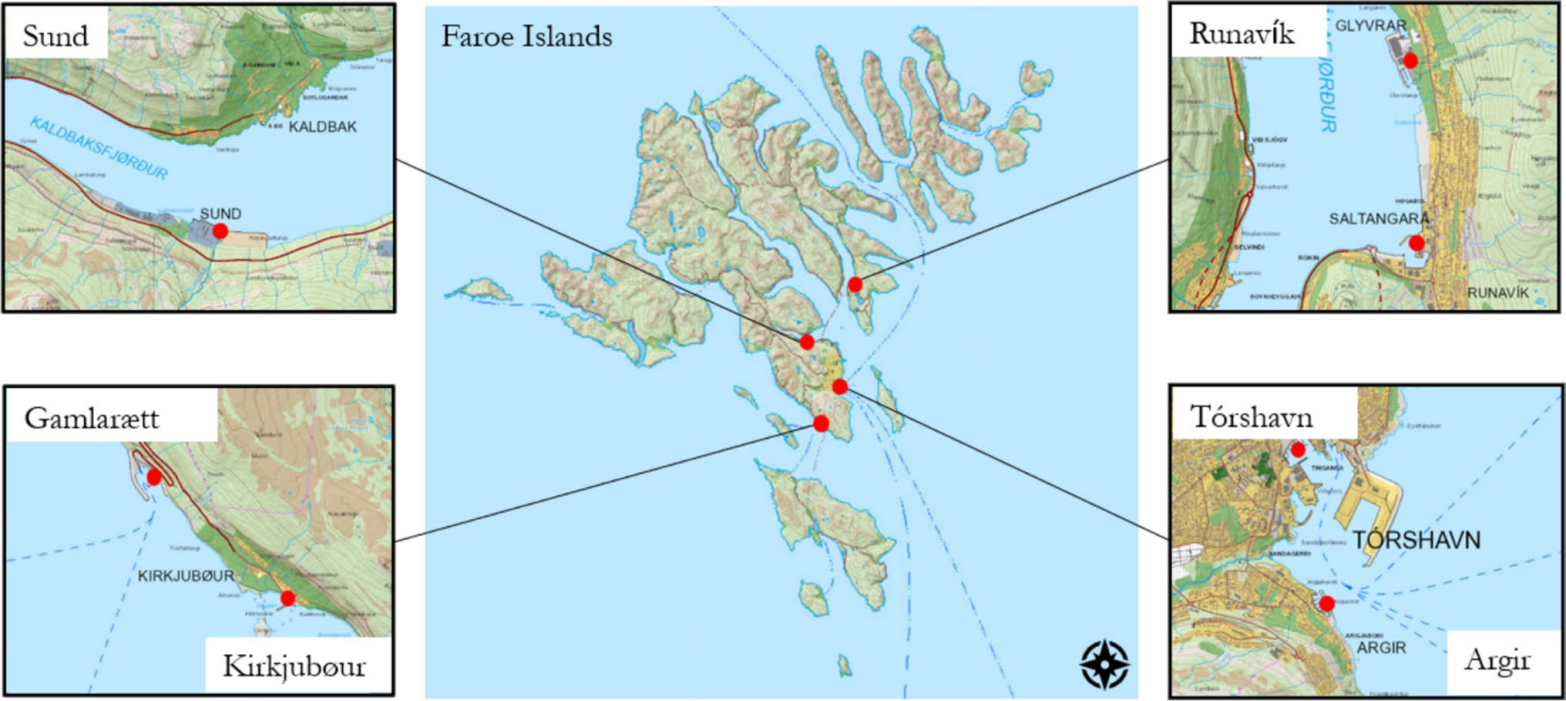
The Faroe Islands and sampling location details of Tórshavn, Runavík, Sund, Gamlarætt, and Kirkjubøur. Red dots indicate sampling locations. Source: kort.foroyakort.fo

The following species were used in this study: shorthorn sculpins (*Myoxocephalus scorpius*), Atlantic cod (*Gadus morhua*), common dab (*Limanda limanda*), saithe (*Pollachius virens*), and common whelk (*Buccinum undatum*). These species populate different tropic levels in the ecosystem and have different metabolisms, feeding, and contaminant uptake pathways (Schultz 2004); (Seibel and Drazen 2007). Samples were collected from the marine waters around the Faroe Islands (Fig. 1) during a 1-week expedition in June 2022.

Tórshavn, Runavík, Sund, Gamlarætt, and Kirkjubøur were chosen as sampling sites. Tórshavn and Runavík Harbors were suspected hotspots of PAH pollution (Fig. 1) through petroleum spills, industrial combustion activities, and heavy ship traffic (Marine Traffic, 2021). The area is located on a harbor edge near an asphalt- and power plant station powered with heavy fuel oil. Gamlarætt hosts the ferry port that connects the main island Streymoy with Hestur and Skopun. Sund and Gamlarætt are therefore also subjects to possible pollution.

Kirkjubøur was chosen as a reference site. It was selected because of less anthropogenic activity compared to other harbors, and the ease of access to the area for sampling.

Sampling was done using stationary traps and fishing rods along the harbor shores. Sampling locations were noted by GPS coordinates (SI.4. Table 2). The fish were identified by their species level according to *Field Guide to Saltwater Fish* by Schultz (2004)) and *Fiskar undir Føroyum* ([Fishes around the Faroe Islands]) by Mouritsen (2007) (SI.2. Table 1). Fish were put down by a punch to the head and then stored in a cooling box with ice for a maximum of 5 h, before arrival to the laboratory where their weights and lengths were measured (SI.4. Table 2). The fish were dissected by scalpel and scissor, and the livers removed and homogenized and put into pre-cleaned labeled glass vials and weighed. When sample preparations were not performed immediately after dissection, the livers were stored at − 80 °C until analysis to prevent enzyme activity.

**Table 1:** Average ∑PAH concentration (±), standard deviation in ng/g (ww) for each species at all sampling sites. Only measurements above DL are included. *Whelk samples were a composited sample of three individual whelks

	*Tórshavn*		*Argir Harbor*		*Runavik*		*Sund*		*Gamlarætt*		*Kirkjubøur (reference)*	
	∑PAH	*N*	∑PAH	*N*	∑PAH	*N*	∑PAH	*N*	∑PAH	*N*	∑PAH	*N*
*Cod*	9 (± 8)	2	11	1	5 (± 5)	7	7	1	-	0	-	0
*Sculpin*	7	1	3	1	-	-	3	1	-	0	5 (± 2)	4
*Whelk*	14 (± 3)	2*	-	0	4	1*	-	0	-	0	-	0
*Common dab*	-	0	-	0	24	1	-	0	-	0	-	0
*Saithe*	-	0	-	0	-	0	-	0	10 (± 3)	3	-	0

**Table 2:** List of detected PAHs and water solubility adjusted from results by Tobiszewski & Namieśnik 2012

PAH		Short name	Molar mass (g)	Water solubility (mg L^−1^ at 25 °C)	Log Kow at 25 °C
Acenaphthylene	Acp		152.19	16	4
Fluorene	Flr		166.22	1.9	4.18
Phenanthrene	Phn		178.23	1.1	4.46
Fluoranthene	Flt		202.25	0.2	8.9
Pyrene	Pyr		292.25	0.13	8.8
Anthracene	Ant		178.23	0.04	4.49

Common whelks were stored for 2–3 h in a cooling box until arrival at the laboratory. Here they were euthanized by fast freezing for 10 min at − 80 °C. After thawing, the visceral complex and gonads of common whelk were removed from their shell (SI.3. Figure 1), then 2 g of the visceral complex from each whelk were weighed and homogenized before immediate extraction.

## Reagents and materials

Acetonitrile (HPLC grade) was purchased from VWR Chemicals (Rosny-sous-Bois, France). Deionized water was dispensed by a Milli-Q system from PURELAB Chorus system (18.2 MΩ cm^−1^, ELGA LabWater, UK). Extraction salts used were magnesium sulfate (anhydrous, ReagentPlus) and sodium chloride (anhydrous, ACS reagent). Magnesium sulfate was obtained from Applichem GmbH (Darmstadt, Germany), and sodium chloride was purchased from VWR chemicals (Rosny-sous-Bois, France). Sodium hydrogen citrate sesquihydrate (ReagentPlus) and sodium citrate dihydrate (FG grade) were purchased from Merck (Germany). The primary and secondary amine (Spera™ PSA) and the dispersive C18 (d-C18) were purchased from Phenomenex (Torrance, California, USA). Naphthalene, anthracene, phenanthrene, flouranthene, chrysene, pyrene, benz(a)anthracene, benzo(a)pyrene, perylene, benzo(g,h,i)perylene, benzo(k)fluoranthene, dibenz(a,h)anthracene, fluorene, benzo(e)pyrene, acenaphthene, acenaphthylene, benzo(b)fluoranthene, and indeno(1,2,3-c,d)pyrene were all obtained from Rathburn (Walkerburn, UK).

Deuterated polycyclic aromatic hydrocarbons: naphthalene-d8, dibenzothiophene-d8, acenaphthene-d10, phenanthrene-d10, pyrene-d10, fluorene-d10, chrysene-d12, benzo(k)fluoranthene-d12, benzo(g,h,i)-perylene-d12, acenaphthylene-d8, anthracene-d10, fluoranthene-d10, benz(a)anthracene-d12, benzo(a)-pyrene-d12, and indeno(1,2,3-c,d)pyrene-d12 were purchased from Rathburn (Walkerburn, UK), and prepared in HPLC-grade isooctane and used as either internal standards or recovery standards (SI.5. Table 6).

## Method optimization

The sample preparation protocol used in this study was adapted from Urban & Lesueur (Urban & Lesueur 2017). The modifications in the sample preparation aimed to adapt the published protocol to a different sample size, to reduce interference from the fatty matrix, and to pre-concentrate the sample extracts to obtain concentrations above instrument detection limits (DLs). Two grams of sample was used to enable analysis of individual fish livers. Furthermore, higher amounts of PSA (172 mg PSA/g sample) were used compared to the original method (30 mg PSA/g sample).

## Extraction and sample preparation

The fish livers and common whelk’s visceral complex and gonads were homogenized with a BOSCH stainless-steel coffee blender (TSM6A013B). Two grams wet weight (ww) of individual fish liver or a composite sample equal to 2 g of visceral complex from the whelk was used for the analysis. The 2 g was randomly subsampled from the blended material and directly transferred to a 10-mL glass centrifuge tube with PTFE caps using a stainless-steel spatula. Two milliliters of Milli-Q water and 4 ml of acetonitrile was added to each sample. The samples were vortexed for 5 min, followed by an addition of a mixture of extraction salts containing 1.6 g magnesium sulfate, 0.4 g sodium chloride, 0.2 g sodium hydrogen citrate sesquihydrate, and 0.4 g sodium citrate dihydrate. The samples were vortexed for an additional 5 min and then centrifuged at 2000 rpm for 10 min. Two milliliters of the supernatant was collected and transferred to another centrifuge tube. A mixture of 260 mg magnesium sulfate, 345 mg PSA, and 45 mg d-C18 was added to the centrifuge tubes for dispersive solid-phase extraction (dSPE) and phase separation. Samples were vortexed again for 5 min and centrifuged for another 10 min at 2000 rpm. Then 1 ml of supernatant was transferred to a new vial and spiked with 60 µl of internal standard mixture consisting of acenaphthylene-d8, anthracene-d10, fluoranthene-d10, benz(a)anthracene-d12, benzo(a)pyrene-d12, and indeno(1,2,3-c,d)pyrene-d12 (SI.5. Table 4a and c) using a 250-µl gas tight syringe (Hamilton Company, USA) and pre-concentrated by evaporation almost to dryness at 50 °C under a gentle stream of nitrogen. The dry extracts were redissolved in 100 µl of acetonitrile and sonicated for 5 min. The extracts were transferred to a 300-µl amber vial with fixed insert for GC–MS measurements.

## Chemical analysis by gas chromatography-mass spectrometry (GC–MS)

The extracts were analyzed by GC–MS using an Agilent 6890N-5975B with electron ionization (EI) at 70 eV. One microliter was injected in splitless injection mode. The oven temperature program was as follows: Initial temperature of 50 °C held for 2 min, increased with 25 °C/min to 315 °C, and held for 8.0 min leading to a total analysis time of 30 min. The capillary column was a 30 m ZB-5 (Phenomenex, 0.25 μm × 0.25-mm film thickness). Helium was used as a carrier gas at 1.1 ml/min. Data was recorded in selected ion monitoring mode (SIM), monitoring 13 selected mass-to-charge ratios (m/z’s) from 128 to 288 Da in 12 retention time intervals across a total analysis time of 27 min. Each m/z was detected with a dwell time of 20 ms. Quantifier ions for PAH analytes are listed in SI.5. Table 3.

## Quality assurance

Before each extraction, samples were spiked with 160 µl of a 8 µg/ml recovery standard mixture consisting of naphthalene-d8, dibenzothiophene-d8, acenaphthene-d10, phenanthrene-d10, pyrene-d10, fluorene-d10, chrysene-d12, benzo(k)fluoranthene-d12, and benzo(g,h,i)perylene-d12 (SI.5. Table 4a and b) to correct for losses during sample preparation.

Quantification of PAHs was done using an internal standard approach. The quantification standard mixture contained 16 individual PAHs in a concentration range from 3 to 180 ng/mL (SI.5. Table 3). The internal standards were used to construct the internal calibration curve and to monitor instrument performance.

The recovery standards were quantified in each sample, and concentrations obtained from the internal standard approach were corrected for less than 100% recovery (SI.5. Table 8 and Eq. 1). The selection of internal standard and recovery standard to represent each analyte was based on compound similarity (SI.5. Table 6). For example, for the two-ring PAH analyte naphthalene, the internal standard was the deuterated two-and-a-half-ring PAH acenaphtylene-d8, and the recovery standard was the deuterated two-ring PAH naphthalene-d8.

For each day of analysis, a quality control sample from a composite liver from three cods was used to monitor intra- and inter-day variations in sample preparation and chemical analysis. Two method blanks were analyzed for every analysis sequence. Spiking and sample re-dissolution were performed with the help of gas-tight glass syringes of 10, 50, and 250 µl (Hamilton Company, USA). The sorbents PSA and d-C18 together with magnesium sulfate were pre-cleaned by packing 5 g of material into a 6-mL glass SPE column and washing with 16 ml of dichloromethane, adding 2 ml each time.

Afterwards, the treated sorbent was left to dry in a glass beaker until total evaporation of dichloromethane. Standard solutions were analyzed in-between samples (usually one standard per four samples). Before every batch, a system suitability test sample was analyzed to assess injection port inertness and GC column performance. The suitability test sample consisted of a mixture of pentachlorophenol, decafluorotriphenylphosphine, benzidine, and 4,4-DDT, each with a concentration of 50 µg/ml.

All peaks were quantified using Masshunter Software (Agilent, USA). Selectivity was confirmed by using at least two qualifier ions and comparison of the relative retention with respect to the corresponding deuterated internal standard. Only PAHs without interference were included. All values were considered for the data treatment, also values below DL and limit of quantification (LOQ). Calculation of DL and LOQ are reported in SI.6.

## Results and discussions

Mean concentration was calculated for each PAH in each species. Hereafter, mean values were summed to describe a total concentration of PAHs measured (∑PAHs). Summed concentrations and standard deviations (±) were compared for each species and sample area in Table 1.

**Fig. 2:**
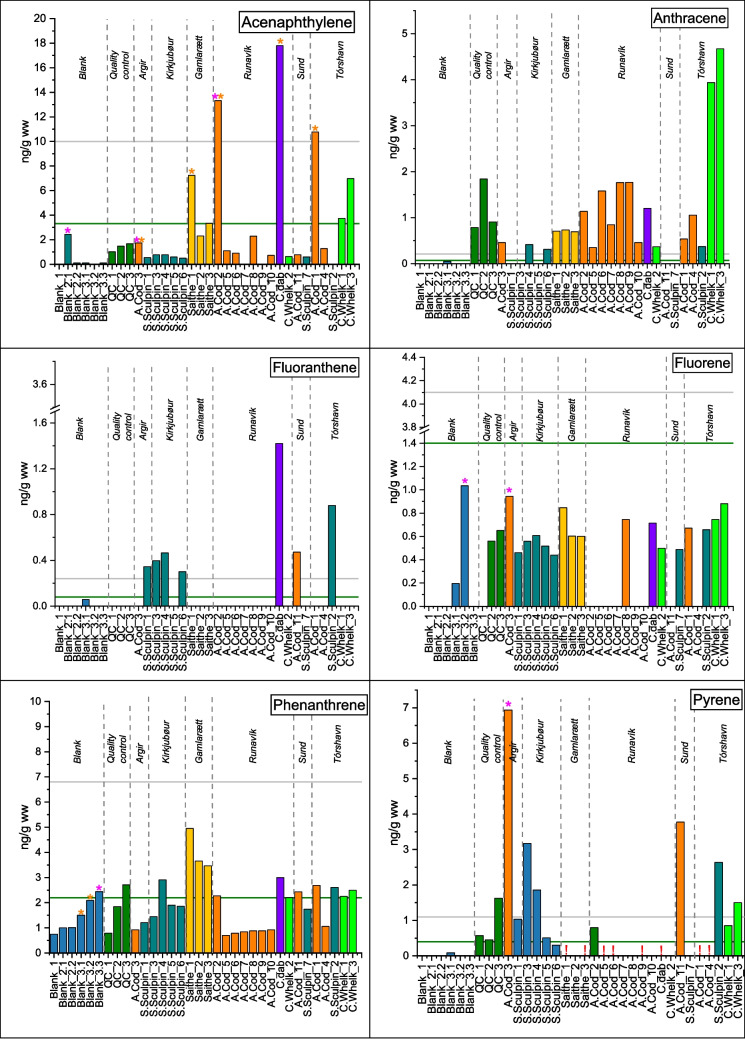
Bar plots of PAH concentrations. The *y*-axis shows concentrations in ng/g (ww), while sample names are shown on the *x*-axis. Species were grouped according to sampling area. The green and gray lines indicate DL and LOQ, respectively. Pink stars show samples with low response of internal standards, and orange stars indicate low response of recovery standards. Exclamation marks show samples for which there was interference on the peak integration for PAH quantification

The highest concentration was found in common dab followed by common whelk, cods, and sculpins (Table 1). Only six PAHs: acenaphthylene, fluorene, anthracene, phenanthrene, fluoranthene, and pyrene, were detected above DL in at least one sample. Fluorene, acenaphthylene, phenanthrene, and anthracene were the PAHs of highest detection frequency (Fig. 2). Highest concentrations for anthracene were found in common whelk in Tórshavn, with an average of 4.3 ng/g (ww), and the average of ∑PAHs found was 11 ± 2 ng/g for the investigated areas (SI.5. Table 7). Phenanthrene was found in highest concentrations in saithe, together with high values of acenaphthylene and phenanthrene (Fig. 2). In a review on the toxicological effects of aquatic animals (Honda & Suzuki 2020), it was observed that the toxic effect is dependent on the aquatic species and the concentration of the individual PAH compounds. Specifically, the findings revealed that the lethal concentration for half a population (LC_50_) for aquatic invertebrates, including water flea species *Daphnia Magna*, was between 4 and 41,000 µg/L. This suggests that these organisms exhibit a relatively high tolerance to the presence of PAHs before experiencing acute toxic effects. The review highlighted the toxicity associated with high molecular weight (HMW) PAHs, particularly pyrene and fluoranthene. These HMW PAHs were found to exhibit lower LC_50_ values of 4 µg/L. In the present study, pyrene was detected in Atlantic cod at concentrations of up to 7 ng/g, while fluoranthene was predominantly identified in shorthorn sculpins at concentrations reaching 1.4 ng/g (Fig. 2). Notably, the current study did not investigate water or sediment concentrations, and direct comparisons with these compartments are challenging. However, in 2002, an environmental survey was done on PAH levels in the Bay of Tórshavn (Dam & Danielsen 2002), mainly on sediments, but also in cod liver. Ten cods were caught at the same harbor around 150 m east of our site called Tórshavn. They detected multiple PAHs, and the levels of PAHs were in a range corresponding to 0.001 to 0.0012 ng/g dry weight. These results were assessed to be an ecological risk that impacted the health of the cods in the area.

**Fig. 3:**
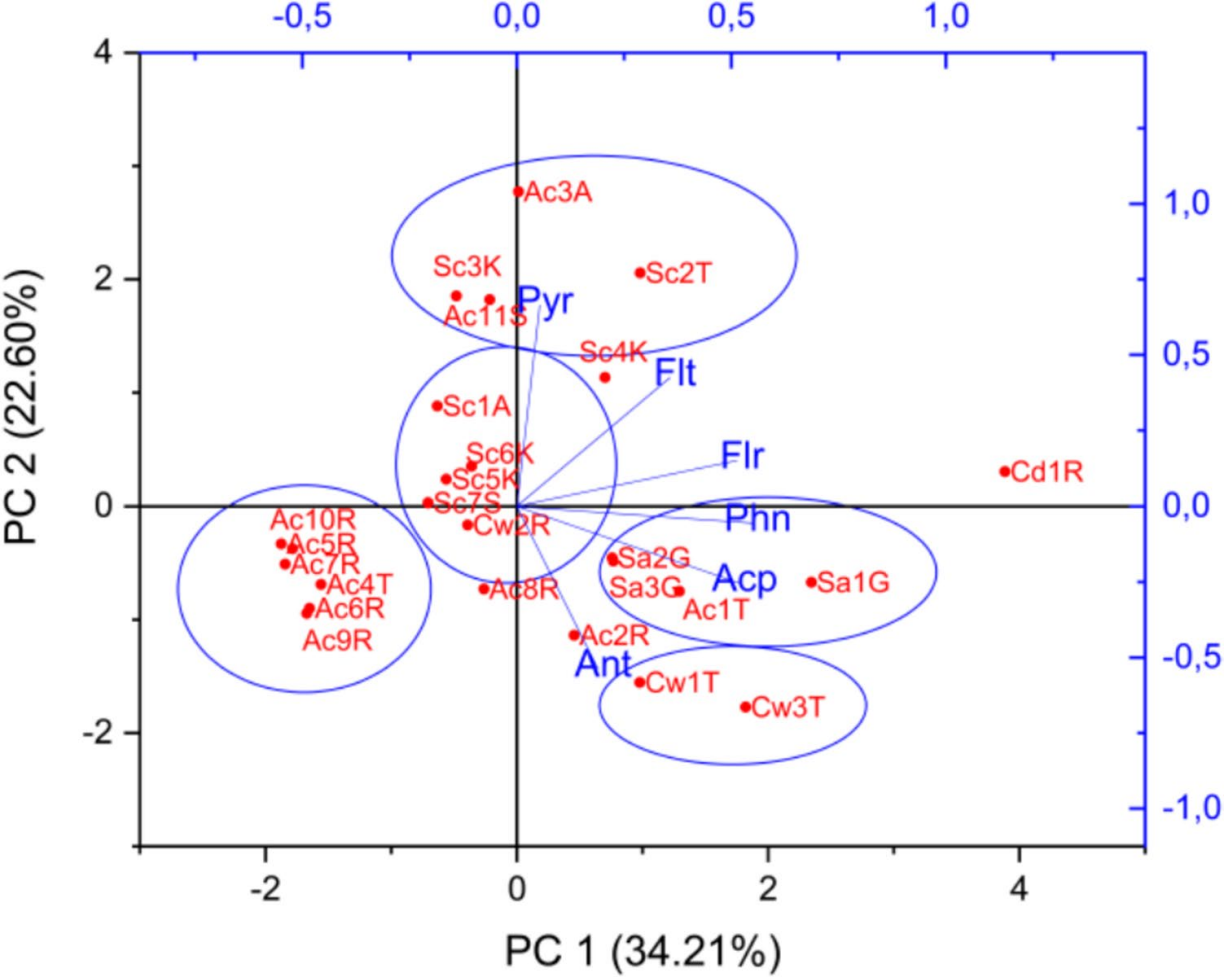
PC1 vs. PC2 biplot of scores and loadings. PAHs variables are shown in blue and samples in red. Samples names are coded with species (Ac, Atlantic cod; Cd, common dab; Sc, sculpin; Cw, common whelk; Sa, saithe; FT, flatfish), followed by the number for the fish individual caught and sample location initials (T, Tórshavn; R, Runavik; A, Argir; S, Sund; K, Kirkjubøur; G, Gamlarætt)

The correlation between PAH concentrations, specimen, and sample site was assessed by principal component analysis (PCA). Samples were normalized to unit sum, and data was mean centered and visualized in a PC1 vs. PC2 biplot in Fig. 3. PC1 shows the variance in PAHs concentration, and PC2 shows the variance in the PAH profiles. Sculpin score values are located at positive PC2, and thus this species has relatively higher concentrations of the HMW PAHs, pyrene, and fluoranthene. Results show that within the benthic species, relatively higher concentrations of the HMW fluoranthene or pyrene (Fig. 2) were found at the suspected hotspots compared to the reference site. The presence of pyrene and fluoranthene in the benthic species can be due to HMW PAHs high affinity to particulate matter that accumulate at the bottom where these species reside, hence the sediment can act as an exposure route of HMW PAHs for these species (Saunders et al. 2022). In contrast, saithe shows correlation with acenaphthylene, a PAH of higher water solubility. Also, our study shows high relative concentrations of acenaphthylene, phenanthrene, and fluorene in the saithe compared to the other fish species. These PAHs are more water soluble and have less affinity for sediment (Tobiszewski & Namieśnik 2012) (Table 2). Therefore, the solubility of acenaphthylene, fluorene, and phenanthrene may be correlated with the high concentration found in saithe that inhabit shallow waters and have their main uptake route through the gills. Common whelks contain relatively high concentrations of anthracene (Fig. 2), which can be due to the selective accumulation of anthracene as reported in the work of Primost et al. (2018) and their low metabolism (Honda & Suzuki 2020).

**Fig. 4:**
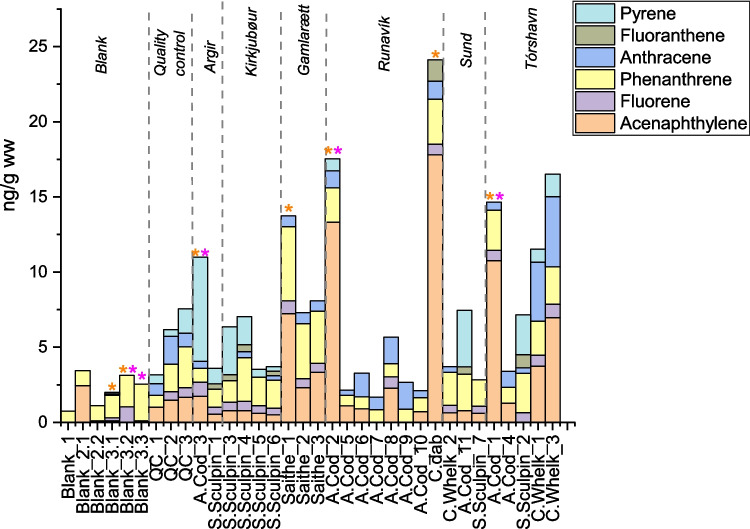
Stacked bar-plot for individual PAH concentrations for all the caught species in all sampling areas. The* y*-axis displays concentration in ng/g (ww) of sample; in the *x*-axis, the different species appear and nested within each sampling area

The cods show varying PAHs concentration patterns, where cods from Sund and Argir have a higher concentration of pyrene compared to Tórshavn and Runavík (Fig. 4). Pyrene is a marker for pyrogenic PAH sources (Jörundsdóttir et al. 2014) and as the sampling site at Sund is located near an asphalt- and power station, the pyrene may have originated from the atmospheric deposition of PAHs from combustion. Cods from Tórshavn Harbor have higher relative concentration of phenanthrene, which indicate a dominant petrogenic PAH pollution source (Pampanin & Sydnes 2013). Furthermore, some cods in Tórshavn and Runavík show some correlation with the HMW fluoranthene and pyrene, while other samples correlate with the three-ring PAHs anthracene and fluorene. Cods are relatively stationary during their juvenile stage and live in the mid to the low depth of the water column typically inside the fjords (Villegas-Ríos et al. 2022). The varying habitats of cods during their juvenile stage make it difficult to use them as a bioindicators for point pollution sources. The correlation between common whelk, saithe, and common dab and the PAHs variables in the multivariate analysis (Fig. 3) is based on a very low number of samples and should therefore be interpreted with caution. It should also be taking into consideration that some meta-parameters like specific fat content and sex have not been investigated.

Likewise, sediment samples from the sampling sites have not been investigated and therefore cannot be used for correlation analyses. However, Tórshavn Municipality has intermittently (2002, 2008, 2014, 2021) measured PAH16 (U.S. EPA) in sediments in and around Tórshavn Harbor (Technical Report, 2021). The results show that the concentration of PAH16 has improved over the years, but the pollution situation at some sites is still in 2021 quite bad and of major concern following Norwegian standards (Bakke et al., 2008). The cods, sculpins, and whelks were caught at the Tórshavn sampling site. The site is located just between the Shipyard and Vágsbotn which were assessed in their study as the most polluted sediment sites. The sediment samples show high concentrations of acenaphthylene, anthracene, fluoranthene, and pyrene which were also identified in this study. The reported sum of PAH16 (U.S. EPA) is 17,000 ng/g at the site closest to ours, i.e., the shipyard.

While the low PAH concentrations and number of samples hampered the accurate identification of its sources using ratios, arguments could be made based on the PAH distribution pattern of acenaphthylene, phenanthrene, and pyrene that makes up a notable part of the total PAH concentration (Fig. 4). Lower concentrations of acenaphthylene were observed at Kirkjubøur compared to Torshavn and Runavík; this compound is found in high concentrations in coal tar (Wise et al. 2010) and in combustion from solid fuel (Ravindra et al. 2008), and has a higher water solubility compared to the other PAHs making the water column easily susceptible to greater concentrations of acenaphthylene. Higher concentrations of acenaphthylene were detected in the demersal species, i.e., cods and saithes (Fig. 2). Torshavn and Runavík are both harbors with a high density of vessel traffic. Thus, vessel traffic may be the main source of acenaphthylene. In 19 out of 25 marine samples, phenanthrene concentrations are higher than anthracene (Fig. 4) which could be an indication of pollution from petrogenic sources. The highest total concentration was found in a common dab from Runavík and in common whelk from Tórshavn together with cods from Runavík and Tórshavn respectively (Table 1). Sculpin was the only species caught at all larger sampling sites (Torshavn, Sund, and Gamlarætt). No clear pollution hotspots were found using the sculpin as a bioindicator. However, our results indicate that PAH concentrations were in general highest in fish and common whelk from Torshavn compared to other sites (see Table 1). In our study, common dab, common whelk, and sculpins, among all the species investigated, correlate with a specific group of PAHs in suspected hotspots.

**Fig. 5:**
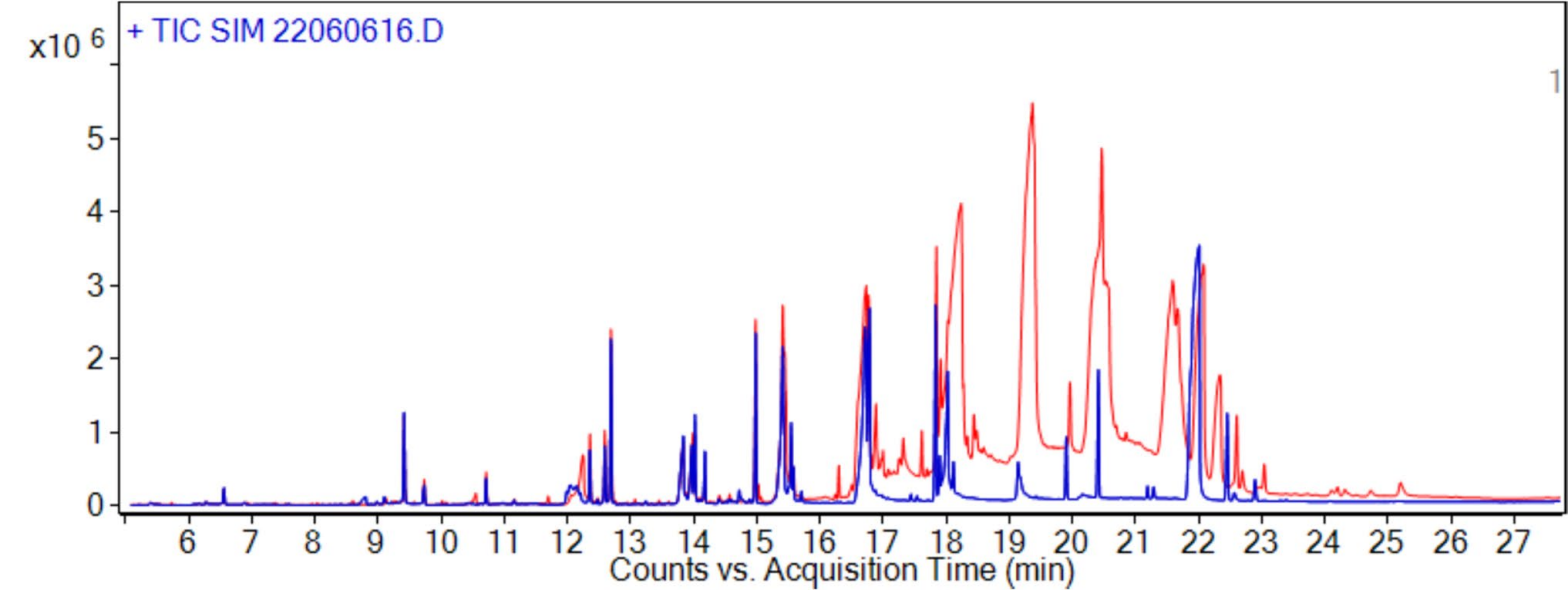
Total ion chromatogram (TIC) of an extract of cod liver worked up using 30 mg PSA/g sample (red line) or using 172 mg PSA/g sample (blue line)

One of the aims of this study was to develop a rapid and cost-effective sample pretreatment method that can be used for fatty marine organism matrices and on site in the Faroe Islands. Using the modified QuEChERS method led to a reduction in C18, C20, C22, and C24 fatty acids (Fig. 5) that originally interfered in chromatograms. Here, DLs ranged between 0.3 and 3.3 ng/g (ww). This range of DLs is onefold higher, compared to the DLs between 0.04 and 0.34 ng/g obtained by Urban & Lesueur (Urban & Lesueur 2017), who investigated salmon high fat content tissue. But also onefold lower than the 1–50 ng/g DLs reported by Forsberg et al. (2011) who used ethyl acetate, acetone, and isooctane (2:2:1 (v/v/v)) as extraction solvent for salmon tissue and 50 mg PSA/g sample (ww). This study included a preconcentration step through evaporation. Evaporation was performed after addition of internal standards, which led to larger variation and sometimes complete loss of the more volatile internal standards. The interference from the fragment ion from the deuterated standards was accounted for when quantifying trace amounts of the analytes. Overall, preconcentration was necessary to detect low concentration in all species, but it reduced method robustness. Whelk samples showed high fat content compared to other samples. For future implementations, using a multi-equilibria approach like cartridge-based SPE instead of the single-equilibria approach of dSPE could improve fat removal efficiency.

## Conclusion

In conclusion, this study demonstrates the feasibility of applying a modified QuEChERS method for the extraction and clean-up of PAHs in the fish livers and marine common whelk. Results reveal that the highest ∑PAHs concentrations, considering all species, were observed in the Torshavn area (12 ng/g), followed by Gamlarætt Harbor (10 ng/g). Conversely, the lowest total ∑PAHs concentrations were detected in Sund and the expected reference site Kirkjubøur (both 5 ng/g). Common whelk exhibited the highest ∑PAHs value (11 ng/g) among the investigated species. While the limited number of samples and the variation between species and sampling sites hinder a significant conclusion on differences between sites, results suggest the potential of marine common whelk and sculpins as bioindicator organisms for PAH pollution in shallow waters of the Faroe Islands, owing to their benthic and stationary characteristics and presence of quantifiable PAHs in the liver.

## Supplementary Information

Below is the link to the electronic supplementary material.

ESM 1 (DOCX 1.96 MB)

## Data Availability

Further data supporting the findings of this study are available in the Supplementary information. Raw data are available from the corresponding author upon reasonable request.
