# Supplementary material for: Screening of polycyclic aromatic hydrocarbons in multiple fish species and common whelk in the Faroe Islands using a modified QuEChERS method
Source: Environ Sci Pollut Res Int. 2025 Oct 21;32(43):24858–68. doi: 10.1007/s11356-025-37058-z (PMC12594697; doi:10.1007/s11356-025-37058-z)
Supplement: Supplementary file 1 — (DOCX 1.96 MB) [file 11356_2025_37058_MOESM1_ESM.docx]

Supplementary information

for

*Screening of polycyclic aromatic hydrocarbons in multiple fish species and common whelks in the Faroe Islands using QuEChERS method*

Ziff Maria Kristensen^a*^, Maria Eckardt Manniche^a*^, Matteo Ottaviani^b*^, Jan H. Christensen^a^, Peter Christensen^a^, Sigurd Christiansen^c^, and Nikoline Juul Nielsen^a^.

^a Department of Plant and Environmental Science, University of Copenhagen, Thorvaldsensvej 40, 1871 Frederiksberg, Denmark^

^b Danish Offshore Technology Centre, Technical University of Denmark, Elektrovej, 375, 2800 Kgs. Lyngby, Denmark^

^c Faculty of Science and Technology, University of the Faroe Islands, Vestara Bryggja 15, FO-100 Tórshavn, Faroe Islands^

^*Shared first authorship.^

**Content**

[1. Laboratory protocol for sample preparation 2](#_Toc199266870)

[2. Species identification 3](#_Toc199266871)

[3. Common whelk dissection 4](#_Toc199266872)

[4. GPS coordinates and meta-parameters 5](#_Toc199266873)

[5. Calibration analytes, recovery and internal standards, and their retention times 6](#_Toc199266874)

[6. DL and LOQ 11](#_Toc199266875)

[7. Systematic errors and method optimization 11](#_Toc199266876)

# Laboratory protocol for sample preparation

QuEChERS on liver (modified from Urban & Leseur 2017):

Steps:

1. Liver is blended in a coffee grinder until visual homogeneity.
2. **2.0 g of liver sample are weighted** into a 10 ml centrifuge tube.
3. Liver is pre-spike with 160 µL of recovery standard and left to fortified for 30 minutes.
4. **2.0 ml Milli Q water** are added the then vials are vortexed for 1 minutes at 400 rpm.
5. **4 ml ACN** are added, and solutions are vortex vigorously for 2 minutes at 400 rpm.
6. **1.6 g MgSO_4_, 0.4 g NaCl, 0.2 g di-sodium hydrogen citrate (DHS), 0.4 g tri-sodium citrate dihydrate (TSCD)** are added to each sample to induce phase separation.
7. Samples are centrifuge for 10 minutes at 2000 rpm.
8. 2.0 ml of the ACN phase (supernatant) are transferred to a centrifuge tube with **250 mg MgSO_4_, 343 mg PSA and 42 mg of C-18.**
9. Samples are vortexed for 5 minutes at 400 rpm.
10. Samples are further centrifuged for 10 min at 2000 rpm.
11. **1.0 mL** of the supernatant are transferred to a 15 mL vial and spike with 60 µL of the internal standard solution.
12. Samples are evaporated almost to dryness (min. half an hour at 50 °C and with a gently nitrogen flow.
13. Samples are redissolved with100 µL of ACN and ultrasonicate for 2 minutes.
14. Samples are transfer to a GC-vial with insert for GC analysis.

# Species identification

| Tabel 1. Species identification and habitat according to Schultz, Ken. 2004. *Field Guide To Saltwater Fish.* John Wiley & Sons, Inc. Hoboken, New Jersey and Mouritsen, Rógvi: *Fiskar undir Føroyum* [translates to: Fishes around Faroe Islands], Føroya Skúlabókagrunnur, 2007 | | |
| --- | --- | --- |
| **Species name** |  |  |
| Shorthorn sculpin (*Myoxocephalus scorpius*) | **Identification:** Sculpins are characterised by having wide bodies with a compressed tail. Bones under the eyes connect to the front of the gills. The eyes are protuberant and located on top of the head.  The patterns are usually mottled as sheen on the pictures to provide camouflage effect.  **Habitat:** Sculpins live in caves and very rocky areas along the shore. They go from just under the surface and up to 180 meters of depth (Schultz, 2003). | 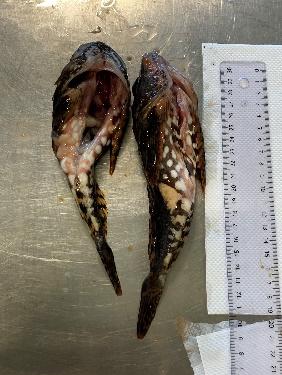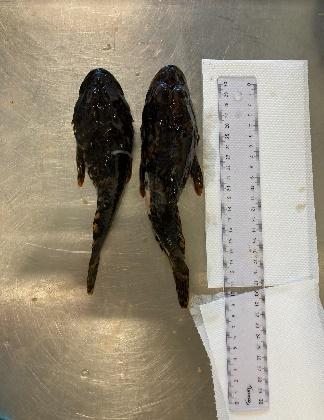 |
| Atlantic cod  (*Gadus Morhua*) | **Identification:**  Atlantic cod are recognised with having three dorsal fins, and two anal fins. None of these have spikes.  The snout has a round shape, and the tail has a characteristic squared shape.  It’s colour vary highly depending on it’s habitat and can cover brown, grey, yellow, red, and greenish colours. It is, however, typically grey white on the underside with light spots covering the rest of the body.  **Habitat:**  Cods prefer cooler water and are found in waters up to 360 meters depth but juveniles stay on shallower water along the coasts. The red-brown colour indicates that the habitat is in the seaweed. | 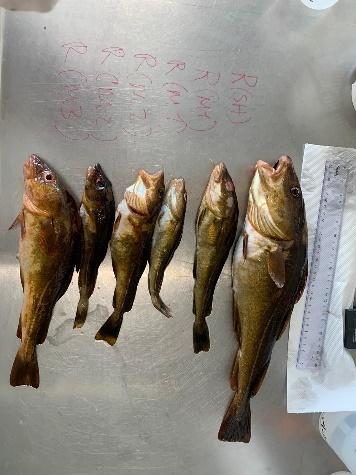 |
| Common dab (*Limanda limanda)* | **Identification:**  The body of flatfish are oval and flat, and the mouth is small. The eyes are on one side of the head making them specialised in living on the bottom.  **Habitat:** Inshore in coastal ocean areas and found on mainly sand-mud bottoms. | Pictures not available |
| Saithe  (*Pollachius virens*) | **Identification:**  Coalfish have very distinguishable jaws with the lower one going beyond the upper one. The tail is compared to the cod shaped as a fork, and the lateral line is straight.  The colours of the coalfish vary from olive green to greenish brown and the bottom is silver coloured.  **Habitat:**  The coalfish is a deep or plagic fish (0-450 meter) and found on rocky bottoms. Typically, the first 3 years (<40cm) the saithe is close to the shores. | 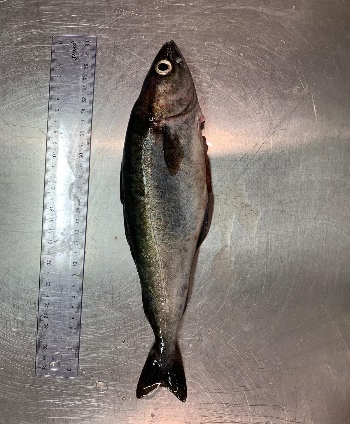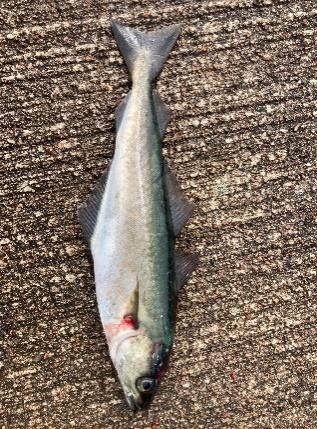 |
| Common whelk (*Buccinum undatum*) | **Identification:**  The Common Whelks were identified as a common whelk, taxonomic species level of *Buccinum undatum*    **Habitat:**  Inshore in coastal ocean areas. | 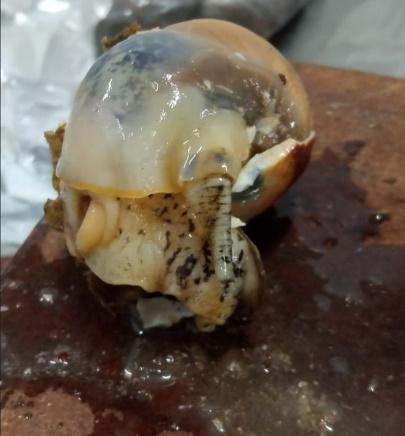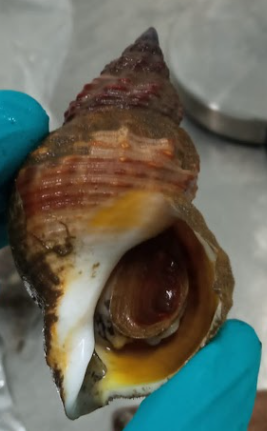 |

# Common whelk dissection

Common Whelks were dissected by removing the visceral complex from the rest of the turgid apparatus. The soft complex composed of kidney, renal conduct, midgut gland, stomach and gonads were removed with a bistoury from the rest of the body. Figure 1 present the species *Littorina littorea,* which is assessed to be comparable to the Common whelk (*Buccinum undatum*) used in this study.


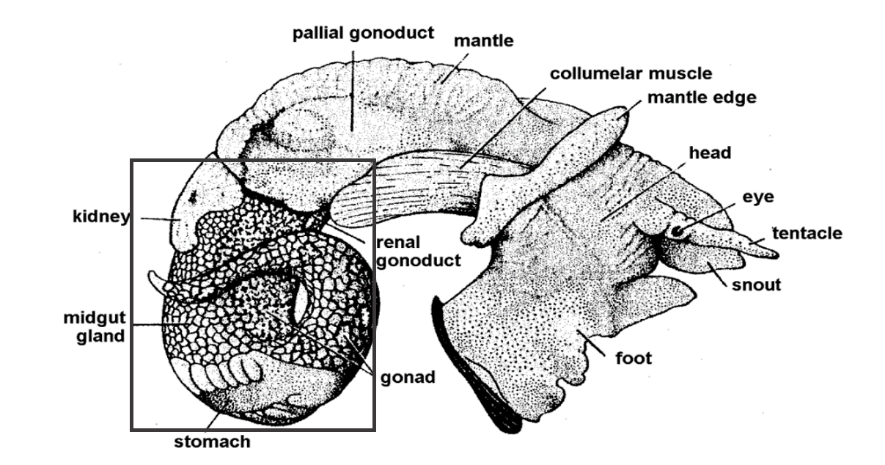


Fig.1 Female *Littorina littorea*. Fretter, V. & Graham, A. 1962

# GPS coordinates and meta-parameters

| **Table 2. GPS coordinates and meta-parameters** | | | | | | |
| --- | --- | --- | --- | --- | --- | --- |
| Location | GPS | Specie | Sample | Weight (*g)* | | Length (cm) |
|  |  |  |  | Total | Liver |  |
| Argir | 61.996205 - 6.768814 | Cod | H | 900 | 5 | 47 |
| Tórshavn | 62.007554 - 6.768262 | Cod | J | 344 | 4 | 30 |
| Runavík | 62.124233 - 6.727676 | Cod | K | 775 | 12 | 40 |
| Runavík | 62.110467 - 6.724089 | Cod | L | 192 | 4 | 26 |
| Runavík | 62.110467 - 6.724089 | Cod | M | 81 | 1 | 20 |
| Runavík | 62.110829 - 6.723695 | Cod | N | 170 | 5 | 24 |
| Runavík | 62.110634 - 6.725136 | Cod | O | 105 | 1 | 21 |
| Runavík | 62.110224 - 6.724196 | Cod | P | 390 | 10 | 30 |
| Tórshavn | 62.008083 - 6.774883 | Cod | Q | 360 | 4 | 33 |
| Runavík | 62.110224 - 6.724196 | Cod | T | 260 | 2 | 30 |
| Sund | 62.049372 - 6.842375 | Cod | E1 | 360 | 4 | 34 |
| Runavík | 62.124465 - 6.727132 | Flatfish | S | 233 | 2 | 27 |
| Gamlarætt | 61.961743 - 6.816729 | Coalfish | U | 210 | 7 | 28,5 |
| Gamlarætt | 61.961743 - 6.816729 | Coalfish | V | 290 | 14 | 32 |
| Gamlarætt | 61.961743 - 6.816729 | Coalfish | X | 205 | 6 | 29 |
| Argir | 61.996205 - 6.768814 | Sculpin | G | 164 | 4 | 20 |
| Tórshavn | 62.008438 - 6.77132 | Sculpin | I | 121 | 1 | 19 |
| Kirkjubøur | 61.952222 - 6.79538 | Sculpin | A1 | 245 | 8 | 25 |
| Kirkjubøur | 61.952222 - 6.79538 | Sculpin | B1 | 230 | 6 | 23 |
| Kirkjubøur | 61.951898 - 6.794763 | Sculpin | C1 | 90 | 2 | 17 |
| Kirkjubøur | 61.951898 - 6.794763 | Sculpin | D1 | 495 | 6 | 31 |
| Sund | 62.049372 - 6.842375 | Sculpin | F1 | 130 | 2 | 20 |
| Tórshavn | 62.008685 - 6.773305 | Common Whelks | H1 | - | - | - |
| Tórshavn | 61.996685 - 6.769 | Common Whelks | G1 | - | - | - |
| Runavík | 62.110634 - 6.725136 | Common Whelks | I1 | - | - | - |

# Calibration analytes, recovery and internal standards, and their retention times

| **Table 3. Target PAH and calibration levels of specific analyte** | | | | | | | | |
| --- | --- | --- | --- | --- | --- | --- | --- | --- |
|  | **MW**  **[Da]** | **Rt**  **[min]** | **Conc.**  **Std 1** | **Conc.**  **Std 2** | **Conc.**  **Std 3** | **Conc.**  **Std 4** | **Conc.**  **Std 5** | **Conc.**  **Std 6** |
|  |  |  | **µg/mL** | **µg/mL** | **µg/mL** | **µg/mL** | **µg/mL** | **µg/mL** |
| **Naphthalene** | 128 | 6.570 | 0.0045 | 0.013 | 0.027 | 0.054 | 0.090 | 0.18 |
| **Anthracene** | 178 | 12.706 | 0.0045 | 0.013 | 0.027 | 0.054 | 0.090 | 0.18 |
| **Phenanthrene** | 178 | 12.619 | 0.0043 | 0.012 | 0.025 | 0.051 | 0.086 | 0.17 |
| **Fluoranthene** | 202 | 14.986 | 0.0041 | 0.012 | 0.024 | 0.049 | 0.082 | 0.16 |
| **Chrysene** | 228 | 17.908 | 0.0038 | 0.011 | 0.023 | 0.046 | 0.077 | 0.15 |
| **Pyrene** | 202 | 15.418 | 0.0036 | 0.011 | 0.022 | 0.044 | 0.073 | 0.14 |
| **Benz(a)anthracene** | 228 | 17.831 | 0.0037 | 0.011 | 0.022 | 0.045 | 0.075 | 0.15 |
| **Benzo(a)pyrene** | 252 | 20.405 | 0.0045 | 0.013 | 0.027 | 0.054 | 0.090 | 0.18 |
| **Perylene** | 252 | 20.546 | 0.0037 | 0.011 | 0.022 | 0.044 | 0.074 | 0.14 |
| **Benzo(g,h,i)perylene** | 276 | 22.849 | 0.0042 | 0.012 | 0.025 | 0.051 | 0.085 | 0.17 |
| **Benzo(k)fluoranthene** | 252 | 19.899 | 0.0034 | 0.010 | 0.020 | 0.041 | 0.069 | 0.13 |
| **Dibenz(a,h)anthracene** | 278 | 22.397 | 0.0038 | 0.011 | 0.022 | 0.045 | 0.076 | 0.15 |
| **Fluorene** | 166 | 10.753 | 0.0040 | 0.012 | 0.024 | 0.048 | 0.080 | 0.16 |
| **Benzo(e)pyrene** | 252 | 20.405 | 0.0035 | 0.010 | 0.021 | 0.042 | 0.070 | 0.14 |
| **Acenaphthene** | 154 | 9.778 | 0.0044 | 0.013 | 0.026 | 0.052 | 0.088 | 0.17 |
| **Acenaphthylene** | 152 | 9.424 | 0.0037 | 0.011 | 0.022 | 0.044 | 0.074 | 0.14 |
| **Benzo(b)fluoranthene** | 252 | 19.861 | 0.0038 | 0.011 | 0.023 | 0.046 | 0.077 | 0.15 |
| **Indeno(1,2,3-c,d)pyrene** | 276 | 22.849 | 0.0035 | 0.010 | 0.021 | 0.042 | 0.070 | 0.14 |

Tab. 3: Calibration curve concentration (µg/ml), molecular weight (MW) and retention timer (RT) details for target PAHs.

| **Table 4a: Concentrations of recovery standards and internal standards in stock solutions** | | | | | |
| --- | --- | --- | --- | --- | --- |
| **Recovery standard stock solution** | **MW**  **[Da]** | **Conc.**  **[µg/mL]** | **Internal standard stock solution** | **MW**  **[Da]** | **Conc.**  **[µg/mL]** |
| Naphthalene-d8 | 136 | 7.03 | Acenaphthylene-d8 | 160 | 8.216 |
| Dibenzothiophene-d8 | 192 | 8.84 | Anthracene-d10 | 188 | 8.593 |
| Acenaphthene-d10 | 164 | 8.47 | Fluoranthene-d10 | 212 | 8.310 |
| Phenanthrene-d10 | 188 | 9.28 | Benz(a)anthracene-d12 | 240 | 7.871 |
| Pyrene-d10 | 212 | 9.19 | Benzo(a)pyrene-d12 | 264 | 8.530 |
| Fluorene-d10 | 176 | 8.62 | Indeno(1,2,3-c,d)pyrene-d12 | 288 | 15.967 |
| Chrysene-d12 | 240 | 7.06 |  | | |
| Benzo(k)fluoranthene-d12 | 264 | 7.34 |  |  |  |
| Benzo(g,h,i)perylene-d12 | 288 | 16.33 |  |  |  |

Tab. 4a: Concentration of stock solutions (µg/ml), molecular weight (MW) for recovery standards and internal standards.

| **Table 4b: Concentrations of recovery standards in calibration solutions** | | | | | | | |
| --- | --- | --- | --- | --- | --- | --- | --- |
|  | **Rt**  **[min]** | **Conc.**  **Std 1** | **Conc.**  **Std 2** | **Conc.**  **Std 3** | **Conc.**  **Std 4** | **Conc.**  **Std 5** | **Conc.**  **Std 6** |
|  |  | **µg/mL** | **µg/mL** | **µg/mL** | **µg/mL** | **µg/mL** | **µg/mL** |
| **Naphthalene-d8** | 6.541 | 0.0070 | 0.021 | 0.042 | 0.070 | 0.14 | 0.21 |
| **Dibenzothiophene-d8** | 12.343 | 0.0088 | 0.026 | 0.053 | 0.088 | 0.17 | 0.26 |
| **Acenaphthene-d10** | 9.720 | 0.0084 | 0.025 | 0.050 | 0.084 | 0.16 | 0.25 |
| **Phenanthrene-d10** | 12.575 | 0.0092 | 0.027 | 0.055 | 0.092 | 0.18 | 0.27 |
| **Pyrene-d10** | 15.384 | 0.0091 | 0.027 | 0.055 | 0.091 | 0.18 | 0.27 |
| **Fluorene-d10** | 10.699 | 0.0086 | 0.025 | 0.051 | 0.086 | 0.17 | 0.25 |
| **Chrysene-d12** | 17.860 | 0.0070 | 0.021 | 0.042 | 0.070 | 0.14 | 0.21 |
| **Benzo(k)fluoranthene-d12** | 19.865 | 0.0073 | 0.022 | 0.044 | 0.073 | 0.14 | 0.22 |
| **Benzo(g,h,i)perylene-d12** | 22.795 | 0.016 | 0.048 | 0.097 | 0.16 | 0.32 | 0.48 |

Tab. 4b: Concentrations of recovery standards in calibration solutions (µg/ml), as well as molecular weight (MW) and retention timer (RT) details for recovery standards.

| **Table 4c: Concentration of internal standards in calibration solutions** | | | | | | | |
| --- | --- | --- | --- | --- | --- | --- | --- |
|  | **Rt** | **Std 1** | **Std 2** | **Std 3** | **Std 4** | **Std 5** | **Std 6** |
|  |  | **µg/mL** | **µg/mL** | **µg/mL** | **µg/mL** | **µg/mL** | **µg/mL** |
| **Acenaphthylene-d8** | 9.400 | 0.32 | 0.32 | 0.32 | 0.32 | 0.32 | 0.32 |
| **Anthracene-d10** | 12.672 | 0.34 | 0.34 | 0.34 | 0.34 | 0.34 | 0.34 |
| **Fluoranthene-d10** | 14.952 | 0.33 | 0.33 | 0.33 | 0.33 | 0.33 | 0.33 |
| **Benz(a)anthracene-d12** | 17.787 | 0.31 | 0.31 | 0.31 | 0.31 | 0.31 | 0.31 |
| **Benzo(a)pyrene-d12** | 20.366 | 0.34 | 0.34 | 0.34 | 0.34 | 0.34 | 0.34 |
| **Indeno(1,2,3-c,d)pyrene-d12** | 22.304 | 0.63 | 0.63 | 0.63 | 0.63 | 0.63 | 0.63 |

Tab. 4c: Concentration of internal standards in calibration solutions (µg/ml), as well as molecular weight (MW) and retention timer (RT) details for internal standard.

| **Table 6. Internal and recovery standards assigned to each analyte PAH** | | |
| --- | --- | --- |
| **PAHs** | **Recovery standard** | **Internal standard** |
| **Naphthalene** | Naphthalene-d8 | Acenaphtylene-d8 |
| **Acenaphthylenes** | Naphthalene-d8 | Acenaphtylene-d8 |
| **Acenaphthalene** | Acenaphthene-d10 | Anthracene-d10 |
| **Fluorene** | Fluorene-d10 | Antrhacene-d10 |
| **Anthracene** | Phenanthrene-d10 | Anthracene-d10 |
| **Phenanthrene** |  |  |
| **Pyrene** | Pyrene-d10 | Floranthene-d10 |
| **Fluoranthene** |  |  |
| **Chrysene** | Chrysene-d12 | Benzo(a)anthracene-d12 |
| **Benzo(a)anthracene** | Chrysene-d12 | Benzo(a)anthracene-d12 |
| **Benzo(B)fluoranthene** | Benzo(k)floranthene-d12 | Benzo(a)pyrene-d12 |
| **Benzo(k)fluoranthene** |  |  |
| **Benzo(a)pyrene** |  |  |
| **Perylene** |  |  |
| **Dibenzo(a,h)anthracene** | Benzo(g,h,i)perylene-d12 | Indeno(1,2,3-c,d)pyrene-d12 |
| **Benzo(g,h,i)perylene** |  |  |
| **Indeno(1,2,3-c,d)pyrene** |  |  |

Tab. 6: Internal standards used for calculating PAH concentrations in samples prior to evaporation and injection to the GC-MS, and recovery standards used to correct for less than 100% recovery during extraction and sample preparation i.e. PAH concentrations in the original samples.

| **Table 7. Summary of single PAH concentrations in individual specimen, where missing values are below of the detection limit (DL)** | | | | | | | | |
| --- | --- | --- | --- | --- | --- | --- | --- | --- |
| Location | **Sample name** | **Species** | **Acenaphthylene** | **Fluorene** | **Phenanthrene** | **Anthracene** | **Fluoranthene** | **Pyrene** |
| Tórshavn | **Cod_1T** | **Cod** | 10.8 | 0.67 | 2.7 | 0.54 | <DL | <DL |
| Runavík | **Cod_2R** | **Cod** | 13.3 | <DL | 2.3 | 1.1 | <DL | 0.79 |
| Argir | **Cod_3T** | **Cod** | 1.7 | 0.94 | 0.92 | 0.46 | <DL | 6.9 |
| Tórshavn | **Cod_4T** | **Cod** | 1.3 | <DL | 1.1 | 1.1 | <DL | <DL |
| Runavík | **Cod_5R** | **Cod** | 1.1 | <DL | 0.70 | 0.35 | <DL | <DL |
| Runavík | **Cod_6R** | **Cod** | 0.90 | <DL | 0.79 | 1.6 | <DL | <DL |
| Runavík | **Cod_7R** | **Cod** | <DL | <DL | 0.85 | 0.85 | <DL | <DL |
| Runavík | **Cod_8R** | **Cod** | 2.29 | 0.75 | 0.88 | 1.8 | <DL | <DL |
| Runavík | **Cod_9R** | **Cod** | <DL | <DL | 0.88 | 1.8 | <DL | <DL |
| Runavík | **Cod_10R** | **Cod** | 0.73 | <DL | 0.92 | 0.46 | <DL | <DL |
| Sund | **Cod_11S** | **Cod** | 0.78 | <DL | 2.4 | <DL | 0.47 | 3.8 |
| Runavík | **Flatsfish_R** | **Flatfish** | 17.8 | 0.71 | 3.0 | 1.2 | 1.4 | <DL |
| Gamlarætt | **Saithe_1K** | **Saithe** | 7.2 | 0.85 | 5.0 | 0.71 | <DL | <DL |
| Gamlarætt | **Saithe_2K** | **Saithe** | 2.3 | 0.60 | 3.7 | 0.73 | <DL | <DL |
| Gamlarætt | **Saithe_3K** | **Saithe** | 3.3 | 0.60 | 3.5 | 0.69 | <DL | <DL |
| Argir | **Sculpin_1A** | **Sculpin** | 0.55 | 0.46 | 1.2 | <DL | 0.34 | 1.0 |
| Tórshavn | **Sculpin_2T** | **Sculpin** | <DL | 0.66 | 2.6 | 0.37 | 0.88 | 2.6 |
| Kirkjubøur | **Sculpin_3K** | **Sculpin** | 0.78 | 0.56 | 1.4 | <DL | 0.40 | 3.2 |
| Kirkjubøur | **Sculpin_4K** | **Sculpin** | 0.79 | 0.61 | 2.9 | 0.42 | 0.46 | 1.9 |
| Kirkjubøur | **Sculpin_5K** | **Sculpin** | 0.60 | 0.52 | 1.9 | <DL | <DL | 0.51 |
| Kirkjubøur | **Sculpin_6K** | **Sculpin** | 0.51 | 0.44 | 1.9 | 0.31 | 0.30 | 0.30 |
| Sund | **Sculpin_7S** | **Sculpin** | 0.60 | 0.49 | 1.7 | <DL | <DL | <DL |
| Tórshavn | **C. Whelk_1T** | **C. Whelks** | 3.7 | 0.75 | 2.2 | 3.9 | <DL | 0.85 |
| Runavík | **C. Whelk_2R** | **C. Whelks** | 0.64 | 0.50 | 2.2 | 0.37 | <DL | <DL |
| Tórshavn | **C. Whelk_3T** | **C. Whelks** | 7.0 | 0.88 | 2.5 | 4.7 | <DL | 1.5 |

Tab. 7: Details on single PAHs concentration (ng/g) and detection limit (DL) and code name of each sample

| **Table 8. Recovery of recovery standards** | | |
| --- | --- | --- |
| **Recovery standard** | **Recovery**  **[%]** | **RSD**  **[%]** |
| Naphtalene-d8 | 1.4 | 20 |
| Acenaphthene-d10 | 15.5 | 25 |
| Fluorene-d10 | 26.1 | 6 |
| Phenanthrene-d10 | 24.3 | 1.6 |
| Pyrene-d10 | 20.9 | 5.6 |
| Chrysene-d12 | 18.0 | 5.8 |
| Benzo(k)floranthene-d12 | 16.8 | 4.4 |

Tab. 8: Recovery of recovery standards and relative standard deviation (%)

**Equation 1**: Calculation of the PAH concentration using the internal standard approach and with correction for less than 100 % recovery estimated using the recovery standards.

$C$_P_$=\frac{Ap\cdot Cis\cdot Srs}{Ais}$

Cp=concentration target PAHs

Ap=area target PAHs

Cis=concentration internal standard

Ais=area internal standard

Srs=recovery of recovery standard

# DL and LOQ

Detection limit (DL) and limit of quantification (LOQ) were calculated from peak areas of target compounds found in the method blanks (MB) containing only internal and recovery standards

DL = MBmean + 3.3 × MBstd

LOQ = MBmean + 10 × MBstd

MBmean and MBstd are the mean and standard deviation of area ratios of target PAHs and their internal standards.

# Systematic errors and method optimization

Results (table 1, figure 3.1) demonstrated high standard deviations and data variation due both small sample size, low concentrations, and low sample homogenization. However, all concentrations detected, also if under DL and LOQ, was included in the results as it was decided suitable for this screening study.

After adding recovery solution to the samples, all samples were pre-concentrated through evaporation. Adding internal standard solution before pre-concentration has caused samples to have lower recoveries within and between each compound, causing a low response for the internal standards and thereby varying quantification and possible overestimation of the more volatile PAHs. Especially the internal standard acenaphtylene-d8 showed non-uniform recovery response, which is expected to be caused from evaporation due to the compound’s low boiling point and vapor pressure. Additionally, internal and recovery standards concentrations in the samples was not adjusted in accordance to the preconcentration step causing a disproportional up-concentration and fragmentation interference from standards compared to analyte.

Overall, pre-concentration was indispensable for detecting low concentration in all species but it reduced method robustness, hence evaporation was considered a systematic error where the overall assess is that a smaller standard deviation and DL could have been achieved by a milder evaporation, a greater number of replicates, a lower concentration of internal and recovery standard and a greater sample homogenization during blending. Suggestions for future method improvements are descaling recovery concentrations for matching analyte concentration, when pre-concentrated.

**References:**

Fretter, V. & Graham, A. 1962. British prosobranch molluscs. Their functional anatomy and ecology. Ray Society, London, 755 pp.

Schultz, K., 2004. Field Guide to Saltwater Fish. John Wiley & Sons, Inc.

Mouritsen, R.: *Fiskar undir Føroyum* [translates to: Fishes around Faroe Islands], 2007, Føroya Skúlabókagrunnur
